# Young University Students’ Academic Self-Regulation Profiles and Their Associated Procrastination: Autonomous Functioning Requires Self-Regulated Operations

**DOI:** 10.3389/fpsyg.2020.00354

**Published:** 2020-03-13

**Authors:** Rafael Valenzuela, Nuria Codina, Isabel Castillo, José Vicente Pestana

**Affiliations:** ^1^Department of Social Psychology and Quantitative Psychology, University of Barcelona, Barcelona, Spain; ^2^Department of Social Psychology, University of Valencia, Valencia, Spain

**Keywords:** autonomy, student, self-regulation, procrastination, youth, well-being

## Abstract

Students’ autonomous self-regulation requires not only self-motivation but also volition or transforming motivation into specific behavioral intentions and following through. Self-regulation includes self-motivation (i.e., goal setting, learning from mistakes) and volitional regulation (i.e., strategic decision making). Furthermore, individual differences, like trait-level perseverance, significantly influence both motivation and volition. Procrastination has been defined as a volitional self-regulation problem, which involves delaying what one had intended to do, in spite of being motivated, and regardless of anticipating adverse consequences. Thus, it is a tendency toward dysregulated behavior - which may stabilize with age - in which subpar self-regulation may lead to procrastination. As a form of dysregulation, procrastination adversely affects young people’s autonomy and well-being by limiting their personal growth. Previous research has confirmed a negative relationship between self-regulation and procrastination. However, more precision is demanded in: (a) examining the intertwined roles of motivational and volitional aspects of self-regulation for procrastination, and (b) distinguishing between different medium, and between medium and high levels of self-regulation. Consequently, it has been suggested that this could be accomplished by means of person-centered analyses, aimed at identifying distinct naturally occurring students’ self-regulation profiles. These profiles would inform differentiated pedagogical approaches to promote self-regulation strategies counteracting procrastination tendencies. We used cluster analysis to identify academic self-regulation profiles and analyze their relationships with procrastination. Participants were 994 young university students from one public and one private university in Catalonia (41.0% men, 58.4% women, and 0.5% non-binary gender). Their age ranged from 18 to 24 years (*M* = 19.69, *SD* = 1.41). Sampling method was intentional, with proportional quotas by sex, academic year, and area of knowledge. The instrument used for data collection incorporated the Short Spanish Self-Regulation Questionnaire (SSSRQ), which includes four dimensions: perseverance, learning from mistakes, goal setting, and decision making; and the Pure Procrastination Scale (PPS), which considers three dimensions: decisional procrastination, implemental delay and lateness. Results obtained by means of cluster analysis distinguished between high and low academic self-regulation profiles, and also between these and two different medium self-regulation profiles, each with specific emphases on particular volitional shortcomings (i.e., weaknesses in decision-making skills and perseverance). These profiles and their relations with procrastination dimensions allow a joint evaluation via structural equation modeling (SEM) to test cognitive motivational strategies (goal setting, decision making, learning from mistakes, and decisional procrastination) together with behavioral aspects (perseverance, implemental delay), considered in the constructs of academic self-regulation and pure procrastination. From this joint evaluation, guidelines are suggested for promoting autonomy among young university students to the detriment of procrastination, thereby – and in accordance with previous research – enhancing students’ well-being and growth.

## Introduction

Developing the capacity to exercise autonomy from a young age has been deeply studied with the self-determination theory (SDT; Ryan and Deci, 2017), as well as with other psychological approaches. According to SDT, autonomous functioning is an indispensable aspect of people’s innate tendencies toward psychological growth, integration, and well-being. Based on the original works of Heider (1958) and De Charms (1968), SDT defines autonomy as the need to self-regulate one’s experiences and actions, entailing a form of functioning associated with feeling volitional, congruent, and integrated (Ryan and Deci, 2017). By definition, autonomy depends on the capacity for self-reflective endorsement of one’s actions, and autonomy need satisfaction depends on behavior being “self-endorsed or congruent” with “authentic interests and values” (Ryan and Deci, 2017, p. 10). Autonomy corresponds to people who willingly comply or wholeheartedly consent to engage in behaviors based on motives which they would also endorse if reflecting upon them autonomously (which do not need to be self-initiated or lack external inputs) (Ryan and Deci, 2006, 2017). Contrarily, people acting against their volition experience incongruence and conflict, thus limiting their well-being.

### Autonomy Need Satisfaction Through Self-Regulation Operations

While autonomy can be considered a formal or abstract need [e.g., “the need for self-regulation of experience and action” (Ryan and Deci, 2017)], self-regulation can be understood as the capacity for exercising the operations which the satisfaction of this need entails. In other words, the need for being or feeling autonomous can only be satisfied through the exercise of self-motivation, volition, and behavioral self-regulation. In this regard, the construct of academic self-regulation has been defined – based on the operations it entails – as the “self-generated thoughts, feelings, and actions for attaining academic goals,” which are “not only important during the development of a skill” but also later during its performance (Zimmerman, 1998, p. 73). Consequently, autonomy need satisfaction in learning would mean being able to autonomously endorse thoughts, feelings, and actions oriented toward reaching goals in academic learning.

From a pedagogical standpoint, we argue that it is relevant to note that self-motivation, volition, and self-regulation of behavior are abilities based on operations, processes, and strategies and, as such, can be developed and improved through supportive conditions of social contexts (Ryan and Deci, 2017). We think that schools where students’ autonomy is aided can help them in deploying self-motivation processes and self-regulation strategies, as it is at this operational level that students can act upon their autonomous functioning and improve it.

Educational research on self-regulation strives to understand proactive efforts in learning, such as personal initiative, resourcefulness, persistence, and responsibility (Zimmerman, 1998; Dewitte and Lens, 2000). These proactive properties of autonomous functioning can only arise if the learner is self-motivated and has competence for self-directed learning; thus, most models of self-regulated learning include these two aspects: self-motivation; and cognitive, metacognitive, and behavioral strategies and processes (Pintrich and De Groot, 1990; Zimmerman, 1995, 1998). Given that these strategies and processes can be learned and improved, self-regulation is “no longer viewed as a fixed characteristic of students but rather as a set of context-specific processes that are selectively used to succeed in school” (Zimmerman, 1998, p. 74).

Zimmerman (1998) argues that students learn best when able to self-regulate major dimensions of their learning (motives, methods, behaviors, environment, company, and time). This occurs in a four-step cycle including: (1) self-evaluation, (2) goal setting and strategic planning, (3) implementation, and (4) outcome evaluation. Analogously to this cyclical model suggested by Zimmerman (1998), recent research assessing self-regulation in university students has found a four-factor structure comprising of perseverance, goal setting, decision making, and learning from mistakes (Pichardo et al., 2014). In this model, learning from mistakes can be understood as a last phase, which may blend into a renewed first phase of goal setting, with both of these phases representing a deliberative motivational dimension of self-regulation occurring before and after performance (Dewitte and Lens, 2000). Whereas the intermediate phase of decision making may represent the cognitive, meta-cognitive, and behavioral aspects of self-regulated learning implementation strategies, in other words, “self-generated thoughts, feelings, and actions” aimed at academic success (Pintrich and De Groot, 1990; Dewitte and Lens, 2000), spanning across initial goal pursuit, planning, avoiding temptations, controlling attention, and dealing with difficulties such as distal goals or fear of failure, while executing goal-oriented behaviors (Steel et al., 2018).

Both the motivational dimension, occurring “before” and “after” the actual practice, and the strategic-operational dimension, happening “during” implementation of behavior are indispensable for the cycle of self-regulation to influence learning outcomes positively; hence, these two dimensions are key considerations in models of self-regulated learning.

Recent research has found that three elements are key to understanding procrastination over time: pacing style (the pace and time at which the person decides, plans, and carries out courses of action), intention-action gap (the times the person fails to enact their own intentions), and goal striving (the effort exerted by the person over time to reach their goals) (Steel et al., 2018). Also, these three elements point in the direction of distinct dimensions having to work well together over time, such as having a timely strategy (which relates closely with constructs like goal setting, decision making, and learning from mistakes), and exerting effort over time (closely related with constructs such as perseverance). Thus, it is possible to expect that perseverance could be linked with goal striving, whereas goal setting, decision making, and learning from mistakes could potentially be linked with pacing style or intention-action gap through, for example, outlining the goals early, with a clear planning and intention-action gap risk assessment. Consequently, intention cannot be realized without strategy, nor will strategy ever yield results without perseverance or goal striving.

Furthermore, it has been argued that self-regulation includes personal characteristics such as “a person’s trait-level perseverance and passion for long-term goals” (Duckworth et al., 2007), sometimes referred to as *grit*, which is argued to be an individual trait and has drawn attention from educational researchers given its consistent prediction of study and achievement outcomes (Wolters and Hussain, 2015). Studies focusing on the relationships between perseverance and preference for long term goals, self-regulation, motivation, and procrastination suggest that perseverance or grit may influence variables such as self-motivation (including, for instance, goal setting and learning from mistakes) and use of cognitive and metacognitive strategies of self-regulated learning (spanning across aspects like goal setting, goal pursuit, and decision making), which in turn may potentially act as mediators between trait-level individual differences in perseverance and outcomes such as learning, achievement, and procrastination (Wolters and Hussain, 2015).

### Procrastination: Volitional Self-Regulation Failure Impairs Growth and Well-Being

The notion that self-regulation entails not only self-motivation and skill but also operations and strategies at the cognitive, metacognitive, and behavioral levels has come partly from studies focusing on volitional dysregulation, called procrastination, a consistent failure to do what one is supposed to do in order to reach their own goals (Lay, 1986). By showing that some students consistently outperformed their peers, regardless of similar motivation and skill, early studies showed that procrastination was indeed a distinct problem. These findings stirred interest in volitional differences in self-regulation, which may account for academic success or failure, that is, differences in dispositions or abilities to “follow through with one’s intentions” (Dewitte and Lens, 2000).

Procrastination has been found to be unrelated to general intelligence (Ferrari, 1991), and procrastinators report a similar number of study intentions than non-procrastinators, but the latter enact more of those intentions (Dewitte and Lens, 2000). Similarly, research has stressed that intention-action gaps, which is implementing a task with delay or being late, are explained by the failure to transform intention into action, but not by intentions, which show no differences between people who procrastinate and who do not (Steel et al., 2018). These antecedents show that volitional problems, such as procrastination, can independently predict variations in critical study outcomes, such as learning or performance, over and above motivation and skill, possibly even mediating between the latter and learning or achievement outcomes (Dewitte and Lens, 2000).

However, procrastination has proven to be a construct with multiple facets (Svartdal and Steel, 2017), including decisional procrastination, linked with subpar planning and decision making (Mann, 1982; Mann et al., 1997); general procrastination, centered on behavioral implemental deLay (1986); and a lateness factor, linked with failing to meet deadlines (McCown and Johnson, 1989).

Furthermore, cyclical models of self-regulation (Zimmerman, 1998) suggest that dysregulations such as poor decision-making skills or low perseverance may both lead to procrastination, low achievement and, thus, to frustration and lower well-being. As a result, procrastination may adversely influence self-evaluations and self-efficacy beliefs, and subsequent motivation and goal setting. Thus, failures in self-regulation are at the core of academic procrastination and pose serious threats to students’ academic achievement and subjective well-being (Steel and Klingsieck, 2016).

These antecedents highlight the pedagogical urgency in supporting young students’ autonomy, growth, and well-being by helping them in overcoming volitional problems, such as the difficulty to transform their intentions or motives into action (Lay, 1986), and the inability to create adequate mental representations of the operations required to successfully tackle the target activity (Dewitte and Lens, 2000). In this regard, students who procrastinate typically face one of two problems. Sometimes, they fail to transform their motivation into volition by failing to convert their goals into precise implemental intentions aimed at enacting specific goal-oriented behaviors, in an adequate and timely manner; they also have been found to choose inadequate implemental intentions, goals, or methods, which grant little support for success. These two aspects, closely linked with goal setting, decision making, and learning form mistakes (which may overlap in time), have also been approached from a longitudinal perspective, in which goal choice and goal pursuit represent two distinct moments: choosing a goal and setting up an initial plan to pursue it (Steel et al., 2018).

## The Present Study

This research combines the interpretations of data offered by the SEM and the cluster analysis. As justified in the following paragraphs – and demonstrated in the Results and Discussion sections – the use of both methods allows the visualization of both the combination between self-regulation and procrastination, and also the identification of how both constructs can manifest in the students. This could potentially offer, in future researches, the possibility of implementing differentiated strategies according to the profile of each individual.

### Full Structural Equation Model for Self-Regulation and Pure Procrastination

The main goal of the present study was to test a structural model of self-regulation and procrastination. Self-regulation and procrastination are closely linked constructs (Steel and Klingsieck, 2016; Steel et al., 2018). Self-regulation includes a dimension of perseverance, as well as self-motivational and strategic aspects (Dewitte and Lens, 2000; Garzón-Umerenkova et al., 2017), whereas procrastination is a self-regulation failure (Steel, 2010), which includes a distinct emphasis on implemental or decisional delays, prompting the failure to meet deadlines, known as lateness factor (Svartdal and Steel, 2017).

However, the pathways through which procrastination may be counteracted by self-regulation are subject to much discussion (Dewitte and Lens, 2000; Svartdal and Steel, 2017). This is especially true given that self-regulation is based on conscious operations and strategies that can supposedly be learned and improved (Pintrich and De Groot, 1990; Zimmerman, 2008), whereas procrastination has been argued to be an irrational deLay (1986), rooted in personal trait-like individual differences (Steel, 2010).

Based on the measurement of three distinct facets of procrastination (implemental delay, decisional procrastination, and lateness; Svartdal and Steel, 2017) we hypothesized a structural model in which procrastination variations were predicted both by a pathway from trait-level perseverance (which is supposed to be difficult to influence, given that it is considered a trait) to implemental delay; and by a pathway from self-regulatory strategies, such as learning from mistakes, goal setting, and decision making (which are supposed to allow for development and improvement) to decisional procrastination.

Self-motivation and use of self-regulated learning strategies have received insufficient empirical research regarding students’ academic achievement (Wolters and Hussain, 2015). Consequently, we asked ourselves if only personal trait-like characteristics (such as perseverance) would account for procrastination variations, or if other distinct aspects of self-regulation, such as self-motivational aspects (goal-setting, learning from mistakes), or even strategic aspects (decision making), could also play specific roles.

The present work hypothesized that self-regulation strategies and processes, such as decision making, goal setting, and learning from mistakes, could be expected to counteract adverse procrastination tendencies in young adults to some extent, by counteracting decisional procrastination over and above the effects of personal trait-level differences, such as perseverance, which were also considered. Even though decisional procrastination has been linked with personal aspects of character (rather than context), explanations have also included aspects like the need for cognition and excessive (metacognitive) clutter, that distracts from decision making (Ferrari et al., 2018). In this regard, self-regulated learning strategies have been found to play critical mediation roles between people’s beliefs about procrastination and decisional procrastination (De Palo et al., 2017), suggesting that, even if not the root of the problem, self-regulation strategies – especially time management – may be key in fostering its prevention. Furthermore, excessive meta-cognition or clutter may be responsible for decisional procrastination, thus, it has been reported that decisional procrastination could be alleviated by attention control (Fernie et al., 2016). Thus, we tested if decision making skills would counteract decisional procrastination through aiding students in keeping their attention under control, that is, focused on the task at hand, given that using cognition to decide and plan actions may help them maintain focus.

Furthermore, we tested if perseverance could positively influence these motivational and strategic aspects of self-regulation and if, in turn, self-regulation strategies could influence decisional procrastination, independent of the effects of trait-level perseverance.

Thus, to analyze particular pathways connecting specific aspects of self-regulation with relevant dimensions of procrastination, we examined three distinct models of self-regulation and pure procrastination via structural equation modeling (SEM).

Taking into account a four-factor self-regulation model (Pichardo et al., 2014; Garzón-Umerenkova et al., 2017) and a three-factor pure procrastination model (Svartdal and Steel, 2017), we hypothesized an integrated seven-factor model with the following characteristics.

The four factors proposed by Pichardo et al. (2014) and validated by Garzón-Umerenkova et al. (2017) seem theoretically appropriate to investigate self-regulation processes in young university students, given that these factors distinguish between a trait-like characteristic (perseverance), a self-motivation dimension of deliberative nature (happening before-and-after action), including goal setting and learning from mistakes, and a strategic dimension, including decision making.

Perseverance has been found to be a stable trait-like characteristic linked with preference for long-term goals, which is expected to influence both student’s motivation and use of self-regulation strategies; these theoretical links, however, have received scarce empirical research (Pintrich and De Groot, 1990; Wolters and Hussain, 2015). Consequently, we hypothesized that perseverance would positively influence self-motivation, such as learning from mistakes (H_1_) and goal setting (H_2_), and the use of self-regulation strategies, such as decision making (H_3_).Furthermore, learning from mistakes (motivational deliberative state happening after action) has been reported to inform subsequent goal-setting and decision making (Zimmerman, 2008); thus we anticipated these paths in the model to be significant and positive: from learning from mistakes to goal setting (H_4_) and decision making (H_5_), as well as from goal setting to decision making (H_6_).

As regards the connections between self-regulation and procrastination dimensions, and in line with theory (Lay, 1986; Svartdal and Steel, 2017), we hypothesized that perseverance would negatively and robustly influence implemental delay (H_7_), given that, in order to be considered an irrational delay rooted in individual differences, significant amounts of the variance of this aspect should be explained through a direct path from trait-like perseverance, and this path should be independent of the strategic dimensions of self-regulation, because, contrarily, the latter can supposedly be learned and improved. As perseverance has been reported to influence not only behavioral dimensions but also cognitive dimensions of self-regulated learning through a greater awareness of and conscious centredness on intended action courses (Pintrich and De Groot, 1990; Wolters and Hussain, 2015), we also anticipated that perseverance would negatively and moderately influence decisional procrastination, given that perseverant students may maintain greater awareness of intended action courses, helping them in finding criteria for decision making from their already set goals and their planning (H_8_).

**FIGURE 1:**
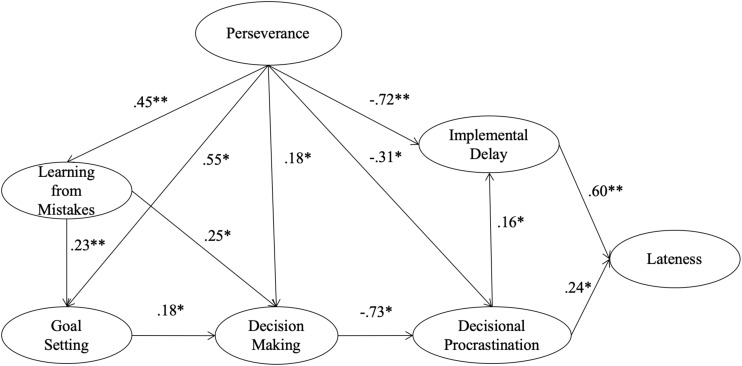
Standardized solution of the hypothesized structural model of the relationship between self-regulation and pure procrastination dimensions. All regression coefficients were significant (*p < 0.05, **p < 0.01). For clarity of presentation, this figure does not include observed variables.

Furthermore, we anticipated that a strategic dimension of self-regulation (decision making) would not only be influenced by motivational dimensions of learning from mistakes (H_4_) and goal setting (H_6_), but that, in turn, it would influence students’ decisional procrastination negatively and robustly (H_9_), thus alleviating implemental delay and lateness problems indirectly (see Figure 1). We expected decision making to influence decisional procrastination negatively and robustly (H_10_).

In regards to mediation effects, we anticipated that a strategic dimension of self-regulation (decision making) would act as a mediator for motivational aspects (learning from mistakes and goal setting) to exert their protective effects against decisional procrastination, given that similar mediations of strategic self-regulation aspects between motivation and learning outcomes have been suggested to be relevant in academic learning (Pintrich and De Groot, 1990).

The goal of this theory-driven model is to inform the discussion about procrastination defined as an irrational delay or volitional self-regulation problem, within which it is relevant to test: (a) pathways connecting self-regulation dimensions (learning from mistakes, goal setting, decision making, and perseverance) with procrastination dimensions (decisional procrastination, implemental delay and lateness); and (b) the potential mediating effect of decision-making strategies between motivational aspects of self-regulation and decisional procrastination.

Thus, this model allows for a discussion of the margins for conscious strategic self-regulated behaviors to counteract procrastination by comparing two distinct pathways connecting both personal trait like characteristics (perseverance) with implemental delay; and self-conscious dimensions of self-regulation (self-motivation, including learning from mistakes and goal setting, and decision making) with decisional procrastination.

### Cluster Analyses

Based on the two main pathways hypothesized to counteract procrastination in our proposed model (perseverance to implemental delay, and strategic aspects of self-regulation to decisional procrastination), we asked ourselves if groupings of students would naturally occur based on their levels of self-regulation dimensions of goal setting, decision making, learning from mistakes, and perseverance (bearing in mind that only the last factor is supposed to be a trait like characteristic, whereas the first three factors are supposed to be possible to be learned and improved).

Prior research has pointed toward difficulty in distinguishing between medium, and between medium and high levels of self-regulation, thus suggesting the use of a person-centered approach to address this issue (Garzón-Umerenkova et al., 2017). In line with this, it has been argued that procrastination-related typologies may address the complex multifaceted nature of procrastination in a more simple way, serving as orientation for pedagogues to inform their practice with empirically based and testable hypotheses (Steel and Klingsieck, 2016).

Thus, we used a person-centered approach (i.e., cluster analysis) to examine different self-regulation profiles, using four self-regulation dimensions (Pichardo et al., 2014; Garzón-Umerenkova et al., 2017) as partition variables (i.e., goal setting, learning from mistakes, decision making, and perseverance), in order to examine distinct naturally occurring academic self-regulation profiles, with distinct shortcomings, among young university students. Regarding self-regulatory strategies, we anticipated that decision making skills would be critical in differentiating between students with medium levels of self-regulation, as strategic aspects of self-regulatory behaviors happen during practice (and not only at an intention level before or after practice, in which procrastinators and non-procrastinators have not been found to diverge). Furthermore, we used emerging profiles to examine between-group differences in procrastination and its dimensions of decisional procrastination, implemental delay, and lateness.

Results from the full structural model and from the cluster analyses are discussed with the goal of informing pedagogues who want to address volitional self-regulation problems in order to support young student’s autonomy and well-being in simplified and group-specific adapted ways.

## Materials and Methods

### Participants

Participants were 994 young university students from one public and one private university in Catalonia (41.0% men, 58.4% women, and 0.5% non-binary gender). Their age ranged from 18 to 24 years (*M* = 19.69, *SD* = 1.41). Sampling method was intentional, with proportional quotas by sex, academic year (first, second, or third), and area of knowledge.

### Procedure

We contacted regular teachers in one public and one private university and asked them to allow researchers to address their students during class. Students voluntarily and anonymously participated by completing standardized 12-minute questionnaires, via an online platform, which was made available to them through a link for the duration of each data collection session.

The ethical requirements of the Ethics Committee of the University of Barcelona (University of Barcelona’s Bioethics Commission, CBUB – Institutional Review Board IRB00003099) were applied to the current study, which meant that additional approval for the research was not required because the data obtained did not involve animal or clinical experimentation. Additionally, this study complies with the recommendations of the General Council of Spanish Psychological Associations (Consejo General de Colegios de Psicólogos), the Spanish Organic Law on Data Protection (15/1999: Jefatura del Estado, 1999), and the Declaration of Helsinki (World Medical Association, 2013).

### Instruments

The instrument used for data collection incorporated two scales. Firstly, the *Spanish Short Self-Regulation Questionnaire* (SSSRQ), proposed by Pichardo et al. (2014), derived from Brown et al. (1999) and validated through Rasch analysis, for Spanish speaking participants, by Garzón-Umerenkova et al. (2017). Evidence for the reliability of this questionnaire has been provided in the original study (i.e., Garzón-Umerenkova et al., 2017) with alphas ranging from 0.71 to 0.81 in its four dimensions and 0.87 in the total questionnaire. The SSSRQ is composed of 17 items divided into four dimensions: goal setting (e.g., “I set goals for myself and keep track of my progress”); perseverance (e.g., “I have a lot of willpower”); decision making (e.g., “I have trouble making up my mind about things”); and learning from mistakes (e.g., “I don’t seem to learn from my mistakes”). The answers are collected on a Likert scale ranging from 1 (not at all like me) to 5 (very much like me).

Secondly, we used the *Pure Procrastination Scale* (PPS: Steel, 2010; Svartdal and Steel, 2017) consisting of 12 items stemming from the General Procrastination Scale (Lay, 1986), the Decisional Procrastination Questionnaire (Mann, 1982; Mann et al., 1997), and the Adult Inventory of Procrastination (McCown and Johnson, 1989), translated into Spanish by Díaz-Morales et al. (2006), which has shown its relevance compared with other measures via factor analysis (Steel, 2010; Svartdal and Steel, 2017). The PPS (Svartdal and Steel, 2017) includes three dimensions: decisional procrastination (e.g., “I delay making decision until it’s too late”), implemental delay (e.g., “I am continually saying ‘I’ll do it tomorrow’.”), and lateness (e.g., “I don’t get things done on time”). Evidence for the reliability of this scale has been provided in the original study (i.e., Svartdal and Steel, 2017) with alphas ranging from 0.83 to 0.87 in the dimensions and 0.92 in the total questionnaire. The answers are collected on a Likert scale ranging from 1 (very seldom or not at all like me) to 5 (very often or very true of me).

### Data Analysis

Statistical analyses were performed with IBM SPPS Statistics version 20 and IBM SPSS AMOS version 24. Data was screened to delete unfinished and unengaged responses (excluding questionnaires reporting only two or less response categories throughout one whole scale). Based on the resulting participant base (*N* = 994) we examined means, standard deviations, and bivariate correlations between study variables. Cronbach alpha coefficients, composite reliability (CR), and extracted mean variance (AVE) were also examined (Table 1).

**TABLE 1:** Descriptive statistics and reliability of study variables (*N* = 994).

	*M*	*SD*	Min	Max	Skew	Kurtosis	Alpha	AVE	CR
*Self-Regulation*	3.27	0.62	1.21	4.83	–0.26	−0.06	0.85	0.57	0.85
Goal setting	3.54	0.79	1	5	–0.52	−0.03	0.83	0.55	0.83
Perseverance	3.18	0.82	1	5	–0.12	−0.42	0.72	0.46	0.72
Decision making	2.91	0.93	1	5	0.00	−0.60	0.78	0.53	0.77
Learning from mistakes	3.45	0.88	1	5	–0.28	−0.54	0.82	0.60	0.82
*Pure procrastination*	2.73	0.76	1	5	0.15	−0.46	0.84	0.55	0.84
Implemental delay	3.25	1.02	1	5	–0.17	−0.82	0.81	0.51	0.81
Decisional procrastination	2.86	0.97	1	5	0.16	−0.61	0.70	0.44	0.70
Lateness	2.08	0.84	1	5	0.67	−0.10	0.68	0.36	0.68

*Range variables: 1–5. AVE = average variance extracted; CR = composite reliability.*

Three different self-regulation and pure procrastination measurement models were compared via confirmatory factor analysis (CFA) in order to proceed to a full structural equation modeling (SEM) phase that would test the theoretical pathways connecting both constructs.

Furthermore, we conducted Ward’s method hierarchical cluster analyses, based on squared Euclidean distances, using dimensions of self-regulation as partition variables, in order to identify naturally occurring groups of students with distinct self-regulation profiles. Based on fusion coefficients and on the proportions of variance explained in partition variables, we chose the four-cluster solution for further analysis. Subsequently, iterative K-means clustering analyses were conducted, using final cluster centers from the hierarchical clustering as initial cluster centers for the iterative method, in order to yield more precise groupings and confirm the stability of the solutions by means of Cohen’s kappa. The final cluster solution is depicted in Figure 1, based on *Z*-values of the final cluster centers in partition variables. Lastly, between-groups differences in dimensions of self-regulation and procrastination were analyzed with ANOVA, as evidence contributing to convergent validity.

## Results

### Descriptive Statistics, Reliability and Bivariate Correlations

Table 1 shows overall mean scores, standard deviations, range, skewness, kurtosis, Cronbach’s alphas, average variance explained (AVE), and composite reliability (CR) for the study variables. With respect to self-regulation dimensions, students’ responses showed that values for the self-motivational aspects of goal setting and learning from mistakes were above the mean value of the questionnaire, while perseverance and the strategic aspect of decision making were under the mean value. In relation to the dimensions of pure procrastination, students’ reported values in lateness were below the mean value of the questionnaire, whereas decisional procrastination and implemental delay were above this value. The internal reliability coefficients for all the scales and subscales were adequate. Cronbach’s alphas and composite reliability ranged from 0.68 to 0.85, whereas AVEs ranged from 0.36 to 0.60 (Table 1). The convergent validity of a construct can be considered adequate when AVE is less than 0.50 but CR is higher than 0.60 (Fornell and Larcker, 1981).

Gender-differences (not tabulated) showed men’s greater tendency toward procrastination and were found for implemental delay (*M*_men_ = 3.38, *SD*_men_ = 0.99; *M*_women_ = 3.18, *SD*_women_ = 1.05; *t* = 2.891, *p* = 0.004), lateness (*M*_men_ = 2.21, *SD*_men_ = 0.85; *M*_women_ = 1.97, *SD*_women_ = 0.81; *t* = 4.194, *p* < 0.001) and overall pure procrastination (*M*_men_ = 2.81, *SD*_men_ = 0.76; *M*_women_ = 2.66, *SD*_women_ = 0.77; *t* = 3.039, *p* = 0.002). However, decisional procrastination showed no gender-differences. Regarding self-regulation, women showed greater scores than men in goal setting (*M*_men_ = 3.37, *SD*_men_ = 0.87; *M*_women_ = 3.59, *SD*_women_ = 0.83; *t* = −3.736, *p* < 0.001) and perseverance (*M*_men_ = 3.01, *SD*_men_ = 0.85; *M*_women_ = 3.23, *SD*_women_ = 0.82; *t* = −2.523, *p* = 0.012), but lesser scores in decision making (*M*_men_ = 3.02, *SD*_men_ = 0.85; *M*_women_ = 2.88, *SD*_women_ = 0.89; *t* = 2.280, *p* = 0.023). Furthermore, no gender-differences were found in learning from mistakes. As a result, overall self-regulation scores showed no gender-differences.

Table 2 shows bivariate correlations between study variables. The four dimensions of self-regulation and the three dimensions of procrastination, respectively, showed robust internal consistency. Because inter-factor correlations are below 0.85 and following Kline (2005) criteria, factor discrimination can be established among the instruments’ dimensions, providing evidence of discriminant validity. Overall, and as expected, self-regulation and its dimensions were robustly and negatively correlated with pure procrastination and its corresponding dimensions.

**TABLE 2:** Bivariate correlations between study variables (*N* = 994).

	1	2	3	4	5	6	7	8
1. *Self-Regulation*								
2. Goal setting	0.75**							
3. Perseverance	0.73**	0.51**						
4. Decision making	0.70**	0.32**	0.30**					
5. Learning from mistakes	0.72**	0.39**	0.36**	0.33**				
6. *Pure procrastination*	−0.70**	−0.50**	−0.60**	−0.53**	−0.39**			
7. Implemental delay	−0.58**	−0.45**	−0.61**	−0.33**	−0.30**	0.84**		
8. Decisional procrastination	−0.63**	−0.39**	−0.41**	−0.64**	−0.35**	0.80**	0.48**	
9. Lateness	−0.48**	−0.37**	−0.42**	−0.29**	−0.31**	0.79**	0.53**	0.44**

**p < 0.01 (2-tailed).

### Three Measurement Models and One Full SEM for Self-Regulation and Pure Procrastination

Prior to analyses concerning the hypothesized structural model, we compared three measurement models (Table 3) to check the factorial structure of the questionnaires. Measurement model 1 comprised seven factors, considering the four-factor self-regulation model (Pichardo et al., 2014; Garzón-Umerenkova et al., 2017), and the three-factor pure procrastination model (Svartdal and Steel, 2017). Measurement model 2 comprised five factors, considering four factors for self-regulation, but one single factor for pure procrastination. Measurement model 3 comprised four factors, considering three factors for pure procrastination, but one single factor for self-regulation.

**TABLE 3:** Three measurement models and one full SEM for self-regulation and pure procrastination.

	CMIN/DF	TLI	CFI	SRMR	RMSEA	90% CI	PCLOSE
Model 1	3.912	0.889	0.903	0.0538	0.054	0.051–0.057	0.011
Model 2	5.622	0.824	0.841	0.0656	0.068	0.065–0.071	0.000
Model 3	9.333	0.683	0.710	0.0806	0.092	0.089–0.094	0.000
Model 1 B	3.846	0.915	0.929	0.0462	0.054	0.050–0.057	0.058
Full SEM	3.798	0.916	0.927	0.0470	0.053	0.049–0.057	0.081

Measurement model 1 showed better fit to the data than models 2 or 3 (Table 3). However, five items did not perform well and were dropped (see Appendix) in line with recommendations from the authors of the SSSRQ questionnaire, arguing that a smaller number of items, reflectively explaining equal or greater amounts of variation in a factor may be seen as an improvement in measurement (Garzón-Umerenkova et al., 2017), if – desirably – at least three items are kept per factor (Bollen, 1989). These theoretically acceptable modifications (deleting five items) produced the final Model 1 B, increasing model fit significantly, as reported in Table 3. Thus, structural equation modeling (SEM) was employed to assess the hypothesized seven-factor structural model (see Figure 1).

The SEM for the hypothesized model (see Figure 1) indicated that perseverance positively and robustly influenced both self-motivational aspects of self-regulation (learning from mistakes and goal setting), as well as the strategic aspect (decision making). Perseverance also showed a negative influence on both decisional procrastination and implemental delay, the latter being more robust. Learning from mistakes positively influenced both aspects of self-regulation (goal setting and decision making). Decision making was also positively influenced by goal setting, although to a lesser extent than by learning from mistakes. Furthermore, decisional procrastination was negatively and robustly influenced by decision making but with a smaller coefficient; perseverance also influenced decisional procrastination negatively. Decisional procrastination positively influenced implemental delay and, lastly, lateness was positively influenced by decisional procrastination and implemental delay, the latter showing a stronger coefficient.

Table 4 shows standardized indirect effects and significance levels of the hypothesized structural model. Perseverance showed the biggest indirect coefficient on lateness, followed by decision making, both negatively influencing lateness. Furthermore, decision making and perseverance also showed significant indirect effects on implemental delay. Decisional procrastination received indirect negative significant effects from perseverance and from both motivational aspects of self-regulation (learning from mistakes and goal setting). Lastly, perseverance showed indirect positive effects on decision making and goal setting.

**TABLE 4:** Indirect effects of the hypothesized structural equation model.

Independent – Dependent variables	Standardized coefficient
Perseverance – Lateness	−0.64*
Learning from Mistakes – Lateness	−0.07*
Goal Setting – Lateness	−0.05*
Decision Making – Lateness	−0.25*
Decisional Procrastination – Lateness	0.10*
Perseverance – Implemental Delay	−0.10*
Learning from Mistakes – Implemental Delay	−0.04**
Goal Setting – Implemental Delay	−0.02*
Decision Making – Implemental Delay	−0.12*
Perseverance – Decisional Procrastination	−0.31**
Learning from Mistakes – Decisional Procrastination	−0.21*
Goal Setting – Decisional Procrastination	−0.13*
Perseverance – Decision Making	0.23**
Learning from Mistakes – Decision Making	0.04**
Perseverance – Goal Setting	0.10**

*p < 0.05; **p < 0.01.

### Cluster Analysis: Self-Regulation Profiles

As a first step in the cluster analysis, we used Ward’s method hierarchical clustering, which combines the two most similar clusters, based on their squared Euclidean distance, starting with *N* = 994 single-person clusters, and ending with one single cluster containing all participants. We observed a clear jump in fusion coefficients (Table 5) when collapsing four groups into three, arguing for the presence of at least four natural groupings.

**TABLE 5:** Fusion coefficients.

Group number	Fusion coefficient
1	2906.02
2	2098.70
3	1827.56
4	1580.98
5	1453.45
6	1356.97
7	1267.25
8	1197.93

As a second step, we applied K-means iterative clustering procedure for a four-group solution, using cluster centers resulting from the hierarchical clustering, as initial centers in a subsequent iterative procedure, taking eight iterations until the largest change in any of the cluster centers was less than 2% of the initial smallest distance. The stability of both solutions was tested through Cohen’s Kappa, which showed an acceptable level (κ = 0.67).

Between-group effects explained 40% of variations in overall self-regulation, 38% in goal setting, 42% in perseverance, 72% in decision making, and 47% in learning from mistakes. Between-group differences also accounted for 31% in overall pure procrastination variance, 39% in implemental delay, 44% in decisional procrastination, and a more modest 17% in lateness.

### Four Self-Regulation Profiles

Table 6 shows final cluster centers for self-regulation dimensions, based on four-group cluster membership: a *de-regulated* cluster, scoring low on all partition variables; a *self-regulated* cluster, scoring high on all self-regulation dimensions; a *low motivation* cluster, characterized by subpar motivation (goal setting, learning from mistakes) and low perseverance; and a *low strategy* cluster, characterized by average motivation (goal setting, learning from mistakes) and medium perseverance, but low decision making.

**TABLE 6:** Final cluster centers on partition variables for four self-regulation profiles.

	De-regulated	Low motivation	Low strategy	Self-regulated	*M*	*SD*
Goal setting	2.70	3.42	3.71	4.17	3.54	0.79
Perseverance	2.43	2.85	3.32	3.93	3.18	0.82
Decision making	2.10	3.54	2.21	3.68	2.91	0.93
Learning from mistakes	2.50	3.27	3.72	4.11	3.45	0.88
*N*	224	232	259	279	994	

**FIGURE 2:**
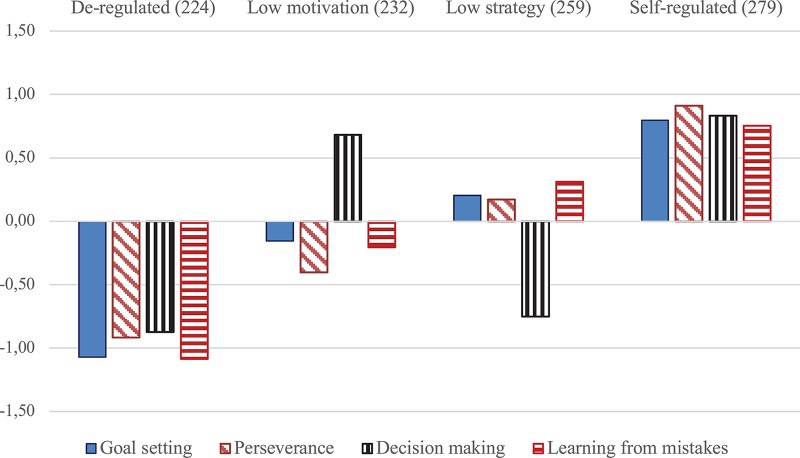
Column diagram depicting *Z*-values in goal setting, perseverance, decision making, and learning from mistakes for four self-regulation profiles.

Figure 2 depicts *Z*-values in goal setting, perseverance, decision making, and learning from mistakes for the four emerging self-regulation profiles. Low motivation students (alongside de-regulated) reported below-average goal setting and perseverance; low strategy students (alongside de-regulated) reported below-average decision making.

**FIGURE 3:**
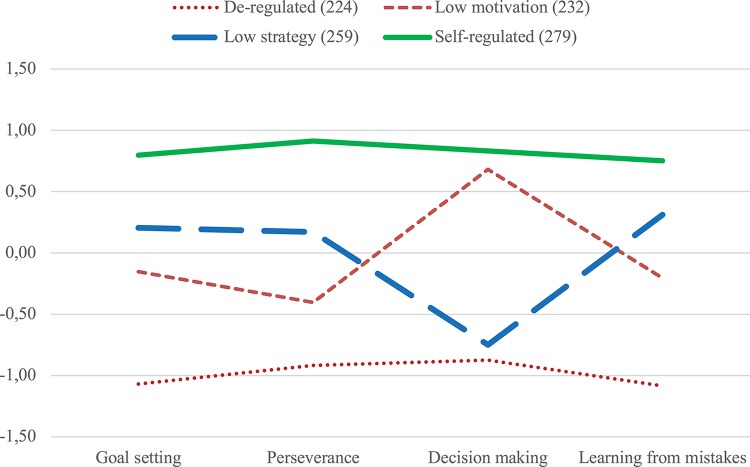
Line diagram depicting four self-regulation profiles based on their *Z*-values in partition variable.

Figure 3 shows a line diagram depicting four self-regulation profiles based on their Z-scores in self-regulation dimensions (partition variables). *Post hoc* tests (using Bonferroni for homogeneous variances and Dunnett’s T for non-homogeneous variances, as judged by Levene’s test) revealed significant differences (*p* < 0.001) in all partition variables between all pairs of groups, except between de-regulated students and low strategy students in decision making (*p* = 0.163). Interestingly, low strategy students scored higher than low motivation students in all partition variables except decision making, where this pattern reversed itself, with decision making, thus, acting as the most important differentiating aspect between groups with medium levels of self-regulation. Lastly, overall self-regulation also revealed significant differences between all groups except between low motivation and low strategy (*p* = 1.000).

### Procrastination Among Four Self-Regulation Profiles

Table 7 shows means in procrastination and its dimensions for each of the four self-regulation profiles. De-regulated students reported the highest mean scores of the four groups in implemental delay, decisional procrastination, lateness, and pure procrastination, but even their reports of lateness were below a neutral score of three points, manifesting a skew in self-reporting. Low motivation students’ mean scores in procrastination dimensions were average, however, slightly above in implemental delay and slightly below in decisional procrastination. Inversely, low strategy students’ mean scores were slightly below-average in implemental delay, but above-average in decisional procrastination.

**TABLE 7:** Four self-regulation profiles and their procrastination dimensions.

	De-regulated	Low motivation	Low strategy	Self-regulated	*M*	*SD*
Pure procrastination	3.45	2.10	2.68	2.83	2.73	0.76
Implemental delay	4.05	2.53	3.33	3.25	3.25	1.02
Decisional procrastination	3.68	2.17	2.55	3.17	2.86	0.97
Lateness	2.62	1.60	2.15	2.08	2.08	0.84
*N*	224	232	259	279	994	

**FIGURE 4:**
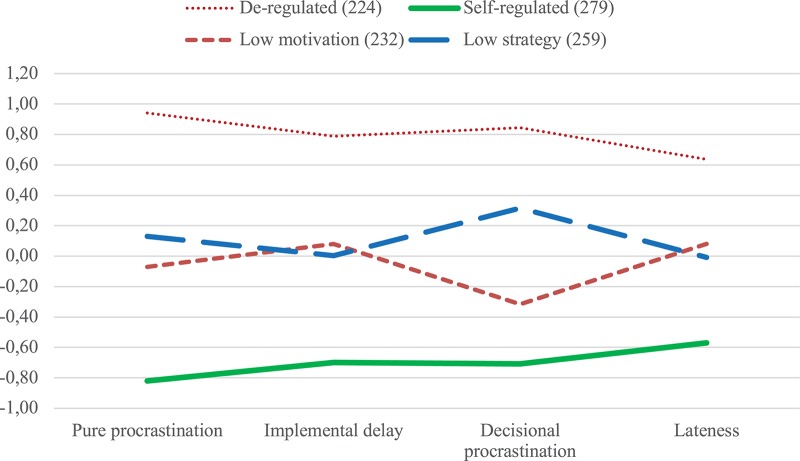
Line diagram depicting four self-regulation profiles based on their *Z*-values in pure procrastination and its dimensions.

Figure 4 shows a line diagram depicting *Z*-values in pure procrastination and its dimensions for the four self-regulation profiles. *Post hoc* tests (using Bonferroni for homogeneous variances and Dunnett’s T for non-homogeneous variances, as judged by Levene’s test) revealed significant differences (*p* < 0.001) in pure procrastination and all its dimensions between all pairs of groups, except between low strategy and low motivation students in implemental delay (*p* = 0.912) and in lateness (*p* = 0.884). Interestingly, low strategy students scored higher than low motivation students in decisional procrastination and overall pure procrastination, marking the relevance of subpar self-regulation strategies such as decisional procrastination for differentiating among groups with medium levels of procrastination.

## Discussion

Pedagogues want to support young university student’s autonomy and well-being by helping them in overcoming volitional self-regulation problems like procrastination, which create frustration, as well as lessening performance, academic achievement, self-efficacy beliefs, and subsequent motivation. Teachers seek simplified and group-specific strategies to understand and counteract students’ procrastination tendencies. However, the discussion about the margins for consciously counteracting implemental delay and decisional procrastination based on self-regulated learning deserves more detailed attention. Consequently, our full SEM model allows for a discussion of the margins for conscious strategic action against procrastination.

According to Model 1B, perseverance positively influences both motivational self-regulation factors (learning from mistakes, goal setting) and strategic aspects (decision making). More perseverant students, therefore, are expected to report higher motivation for self-regulated learning. In turn, self-motivational aspects also exert significant positive effects on the strategic aspect of decision making.

Furthermore, comparing between resulting self-regulation clusters, perseverance, and motivational aspects (learning from mistakes, goal setting) increased or decreased concomitantly, whereas decision making manifested high or low levels, independent of the levels of self-motivation and perseverance of that cluster. It was the strongest variable to differentiate between medium-level profiles of self-regulation.

The two most important paths connecting self-regulation and procrastination dimensions were the strong negative path from perseverance to implemental delay, and the strong negative path from decision making to decisional procrastination. The first path serves as evidence that more perseverant students may remain more aware of their goals, preventing them from slipping into irrational implemental delays. However, as this path roots on personal characteristics, it is still worthwhile to explore alternative paths to counteract procrastination through self-regulation strategies.

The second path suggests that decisional procrastination (and its contribution to implemental delay and lateness) may be prevented via strategic decision-making skills, which could be improved consciously. This strategic dimension of self-regulation seems to be acting as a mediator between self-motivational antecedents (learning from mistakes, goal setting) and decisional procrastination. Noteworthy is the fact that the strongest predictor of decision making in the model was learning from mistakes, which influenced both goal setting and decision making positively. In this regard, we want to stress that learning from mistakes and decision making are two dimensions of self-regulation which cannot be detached from autonomy, given that their operations rest on it.

To a lesser extent, decisional procrastination was also directly influenced by perseverance and, in turn, decisional procrastination influenced implemental delay positively. Furthermore, the lateness factor received the most robust causal path from implemental delay. These findings suggest that decisional delays may be the cause of lateness, and also serve as grounds for implemental delays; the strongest lateness predictor, however, was still perseverance-related implemental delay.

The model supports the argument that strategic decision-making skills may act as a partial mediator between motivational aspects of self-regulation and decisional procrastination. It also supports the belief that that both motivational and strategic self-regulation operations may act as partial mediators between perseverance and decisional procrastination, allowing for the discussion of potential conscious and intentional self-regulation strategies to counteract procrastination tendencies and their adverse impact on young university students’ autonomy, growth, and well-being.

Furthermore, the present study provides a person-centered analysis of four distinct students’ self-regulation profiles and their associated procrastination. By means of a cluster analysis we identified a self-regulated and a de-regulated group, respectively, with the highest and the lowest scores on all self-regulation dimensions. Furthermore, we also differentiated two medium level self-regulation groups, whose main diverging factors were distinct shortcomings, respectively, in perseverance and decision making.

Of the two medium self-regulation groups, low strategy students scored above average in perseverance and in motivational aspects of self-regulation (learning from mistakes, goal setting), but considerably below average in the strategic dimension of decision making (as low as de-regulated students). Consequently, they also scored higher in decisional procrastination than did low motivation students. As a result, notwithstanding the optimistic outlook of their higher motivation (learning from mistakes, goal setting), low strategy students’ overall pure procrastination scores were higher than their low motivation peers’. In contrast, low motivation students (notwithstanding their lower motivation and perseverance), scored similarly to their low strategy peers on implemental delay and lateness, and lower on overall pure procrastination. These findings stress the notion that procrastination is not a problem of motivation but rather a failure of strategic volitional self-regulation.

Combining the interpretations of the structural model (Model 1B) and cluster analyses, we provide an account of the pathways connecting self-regulation dimensions (learning from mistakes, goal setting decision making, perseverance) with procrastination dimensions (decisional procrastination, implemental delay, lateness). This account enables the discussion about particular shortcomings in self-regulation operations (perseverance versus strategic and motivational aspects), linking these with specific facets of procrastination (implemental and decisional delays). We understand that irrational delay occurs when conscious volitional self-regulation operations fail; thus, we argue that providing young students with autonomy support and rationales, aiding their design of autonomous strategies (including abilities for promoting both their motivation and decision making skills), may help them maintaining conscious goal centeredness and strategy engagement, thus, preventing the irrational slip into procrastination.

It has been discussed that procrastination may be linked with personal trait-like characteristics such as perseverance and low ability for emotional regulation, which producer an irrational delay and thus leave little margin for other causal explanations or even for counteracting the behaviors through conscious behavioral regulations. In this regard, it may be true that – in retrospective – procrastination is always rooted in irrational dysregulation, but the question of whether it can be consciously prevented – in our opinion – still holds sway.

It has been argued that procrastination may be promoted by sub-par mental representations of required or viable operations necessary for target activities. Could this mean that there is space for strategies of self-regulated learning to gain terrain on this potential realm of irrational delay, through increasing and maintaining conscious centeredness on adequate goals and strategies? Furthermore, it has been reported that procrastination tendencies may interact with contextual-factors such as teacher autonomy support or control (Codina et al., 2018). Thus, supporting young university students’ autonomy, by facilitating self-regulation, may promote timely goal setting, initial goal pursuit, decision making, planning, and goal striving (Steel and Klingsieck, 2016; Steel et al., 2018). We argue that increased goal centeredness and awareness of self-regulation strategies may, therefore, potentially counteract procrastination tendencies; future research is needed, however, to further address this point.

An important issue to put to test by future studies would be if greater awareness of the self-motivational and strategic dimensions of self-regulation can be fostered through the support of young students’ autonomy, and by facilitating their psychological processes of learning from mistakes, goal setting, and decision making. Given that making successful strategic decisions requires careful self-examination and adequate mental representations of tasks and person-context interactions, we argue that facilitating students’ self-awareness and enhancement of their own motivations, abilities, and strategies, may prevent them from slipping into the realm of irrational delay, by facilitating the inverse processes of autonomous self-regulation.

## Final Remarks

The four emerging self-regulation profiles and the seven-factor structural model connecting self-regulation and procrastination suggest that low strategy students (low in decision making) may benefit from interventions that help them develop adequate mental representations of tasks and person-context interactions, and consider their autonomous goals and past experiences. These students carry above-average self-motivation but below-average strategic self-regulation, thus separating themselves from self-regulated students in regard to general self-regulation and procrastination.

Findings also suggest that low motivation students’ above average reports of decision making may be insufficient for their academic adjustment, given that these students are also characterized by below average goal setting and learning from mistakes, and these variables exert significant indirect effects on decisional procrastination through decision making. Low motivation students’ self-regulation shortcomings stemmed greatly from their below average perseverance and self-motivation, thus these students could benefit from support in building up a repertoire of autonomous pursuits, which they value personally, and which depend on autonomous reflection upon previous goal-oriented experience (learning from mistakes).

Cyclical models of self-regulation suggest that, in order to avoid frustration and a consequent decrease in subsequent motivation, it would be of cardinal importance that these students accomplish their autonomous goals, thus, adequate guidance or support for the optimal selection and scaffolding of initially attainable goals may be key to increased academic success among these students.

## Data Availability Statement

The datasets generated for this study are available on request to the corresponding author.

## Ethics Statement

The Ethical requirements of the Ethics Committee of the University of Barcelona (University of Barcelona’s Bioethics Commission, CBUB – Institutional Review Board IRB00003099) were applied to the current study, which meant that additional approval for the research was not required because the data obtained did not involve animal or clinical experimentation. Additionally, this study complies with the recommendations of the General Council of Spanish Psychological Associations (Consejo General de Colegios de Psicólogos), the Spanish Organic Law on Data Protection (15/1999: Jefatura del Estado, 1999), and the Declaration of Helsinki (World Medical Association, 2013). The patients/participants provided their written informed consent to participate in this study.

## Author Contributions

RV contributed to the conception and design of the study, organized the database, performed the statistical analysis, and wrote the first draft of the manuscript. NC conceived and designed the research, drafted the work, and revised it critically for important intellectual content. IC was responsible for supervising the analysis and interpretation of data gathered, revising it critically. JP contributed to the conception and design of the study, wrote sections of the manuscript, and revised it critically. All authors contributed to manuscript revision, read and approved the submitted version.

## Conflict of Interest

The authors declare that the research was conducted in the absence of any commercial or financial relationships that could be construed as a potential conflict of interest.

## Appendix

The following are the scale construction decisions made in the research:

(1) Implemental delay item: “I often find myself performing tasks that I had intended to do days before” was dropped, given that its squared multiple correlation was <0.3 (= 0.022) and its standardized regression weight on its factor was <0.4 (= 0.15); as a result, the alpha for implemental delay increased from 0.74 to 0.81.
(2) Decision making item: “I have so many plans that it’s hard for me to focus on any one of them” was dropped, given that its squared multiple correlation was <0.3 (= 0.117), and its standardized regression weight on its factor was <0.4 (= 0.34). One more decision making item was dropped: “Little problems or distractions throw me off course,” given that its standardized residual covariance with two other items was >3.0 (= 6.348 with perseverance item: “I get easily distracted from my plans”; and 4.695 with goal setting item: “I have trouble making plans to help me reach my goals.” Worth is noting that in the Spanish version “off-course” is translated as “disoriented,” which might lead attention away from the notion of “action course” – as relevant in decision making – and toward the notion of “chaos and confusion,” consequently, interacting with perseverance and goal setting items. As a result of deleting these two items the alpha for decision making increased from 0.76 to 0.78.
(3) Similarly, goal setting item: “If I make a resolution to change something, I pay a lot of attention to how I’m doing” was dropped, given that its standardized residual covariance with a perseverance item was >3.0 (= 3.953 with perseverance item: “I have a lot of will power”). Worth is noting that in the Spanish version the phrasing “resolution to change something” was translated as “determination of doing something,” subtracting the notion of “change,” thus, reflecting on an aspect less linked with self-motivation and more with strategy.
(4) Lastly, another goal setting item: “I usually keep track of my progress toward my goals” was dropped, given that the absolute value of its standardized residual covariance with a decision making item was >3.0 (= −3,636 with item: “I have trouble making up my mind about things”). Worth is noting that the Spanish version of this goal setting item includes the notion of “study goals,” not just “goals”; and that this decision making item replaces the notion “make up my mind” – unavailable in Spanish – with the phrase: “decide about things,” thus interacting with goal setting dimension.
